# Comparative genomics reveals selective distribution and domain organization of FYVE and PX domain proteins across eukaryotic lineages

**DOI:** 10.1186/1471-2164-11-83

**Published:** 2010-02-02

**Authors:** Sumana Banerjee, Soumalee Basu, Srimonti Sarkar

**Affiliations:** 1 Department of Biological Sciences, Indian Institute of Science Education and Research, Kolkata, Mohanpur, Nadia 741252, West Bengal, India; 2 Department of Biotechnology, School of Biotechnology, West Bengal University of Technology, BF 142, Salt Lake, Kolkata 700064, India

## Abstract

**Background:**

Phosphatidylinositol 3-phosphate is involved in regulation of several key cellular processes, mainly endocytosis, signaling, nuclear processes, cytoskeletal remodelling, cell survival, membrane trafficking, phagosome maturation and autophagy. In most cases effector proteins bind to this lipid, using either FYVE or PX domain. These two domains are distributed amongst varied life forms such as virus, protists, fungi, viridiplantae and metazoa. As the binding ligand is identical for both domains, the goal of this study was to understand if there is any selectivity for either of these domains in different taxa. Further, to understand the different cellular functions that these domains may be involved in, we analyzed the taxonomic distribution of additional domains that associate with FYVE and PX.

**Results:**

There is selectivity for either FYVE or PX in individual genomes where both domains are present. Fungi and metazoa encode more PX, whereas streptophytes in viridiplantae encode more FYVE. Excess of FYVE in streptophytes results from proteins containing RCC1and DZC domains and FYVE domains in these proteins have a non-canonical ligand-binding site. Within a taxonomic group the selected domain associates with a higher number of other domains and is thus expected to discharge a larger number of cellular functions. Also, while certain associated domains are present in all taxonomic groups, most of them are unique to a specific group indicating that while certain common functions are discharged by these domains in all taxonomic groups, some functions appear to be group specific.

**Conclusions:**

Although both FYVE and PX bind to PtdIns(3)P, genomes of different taxa show distinct selectivity of encoding either of the two. Higher numbers of taxonomic group specific domains co-occur with the more abundant domain (FYVE/PX) indicating that group-specific rare domain architectures might have emerged to accomplish certain group-specific functions.

## Background

Phospholipids, far from being mere structural units of various bio-membranes, play important roles in several physiological processes [1-3]. For example, phosphoinositides (PIs), which are the phosphorylated derivatives of phosphatidylinositol (PtdIns), are components of different cellular membranes. There is selective enrichment of particular PIs on the surface of specific organelles [1,2]. At these locations they function as spatial signals for the targeting of specific effector proteins from a cytosolic location to the membrane periphery. The targeting of these effectors to specific membranes is mediated by their lipid-binding domains that are capable of recognising a specific PI [4]. Once at the intended cellular locations, the effectors participate in multiple cellular functions such as signaling, nuclear processes, endocytosis, cytoskeletal remodelling, cell survival, membrane trafficking, phagosome maturation and autophagy [4-6]. Thus PIs play a central role in many crucial cellular events.

Seven different varieties of PIs are formed when PtdIns undergoes differential phosphorylation at the 3-, 4- and 5- hydroxyl groups of its myo-inositol moiety [1]. Phosphatidylinositol 3-phosphate {PtdIns(3)P} is one of these seven. PtdIns(3)P localizes mainly to endosomal membranes [7], but has also been detected within the nucleus [8]. This lipid regulator plays a central role in endocytosis and has also been implicated in signaling events as well.

In most cases PtdIns(3)P-interacting proteins bind to this lipid by using either of two domains, FYVE or PX [9-11]. However there are reports of C2 and PH domains that are also capable of binding to this lipid [12,13]. The FYVE domain is a specific type of zinc-finger motif and is named after the four proteins in which it was initially identified (**F**ab1p, **Y**OTB, **V**ac1 and **E**EA1) [14]. It is 60-70 amino acids long and is rich in cysteines. Three conserved stretches of amino acids are the hallmark of this domain: the WxxD motif at the N-terminal end, followed by the R(R/K)HHCR and finally RVC towards the C-terminus [15-17]. These three motifs, along with other cysteines form the PtdIns(3)P binding pocket. Additional non-specific electrostatic interactions as well as hydrophobic interactions, via a membrane-insertion loop that penetrates the membrane, stabilize the binding of this domain to PtdIns(3)P containing membranes [10]. In addition, multimerization of FYVE domain has been reported to augment membrane binding [18,19]. In contrast to FYVE domains, there is very little sequence similarity between the different PX domains, which are ~130 amino acids in length [11]. However, these diverse sequences fold to adopt a common three dimensional structure with two conserved elements: (i) the PxxP motif capable of interacting with SH3 domain; (ii) the basic residues that constitute the PI binding pocket [10]. Similar to the FYVE domain, additional hydrophobic (also via membrane insertion loop) and electrostatic interactions stabilize the binding of PX domains with membranes. Several PX domain-containing proteins also contain dimerization domains, such as the coiled-coil domain in case of sorting nexins [20,21]. Thus oligomerization is also likely to play a role in increasing the affinity of PX domain for the membrane. Therefore, although FYVE and PX domains have very different structures, they bind to the same ligand PtdIns(3)P, and this protein-ligand binding is stabilized by similar electrostatic and hydrophobic interactions [10]. In addition, in both cases oligomerization contributes to ligand affinity.

Although a majority of PX domains bind to PtdIns(3)P [11,22], there are reports of PX domains binding to PtdIns(3,4)P_2 _[23], PtdIns(4)P [24], PtdIns(4,5)P_2 _[25], PtdIns(3,5)P_2 _[26] and PtdIns(3,4,5)P_3 _[27]. Similarly even though FYVE is considered to be very specific for PtdIns(3)P, the FYVE of EEA1 has been shown to be capable of binding to PtdIns(5)P [28] and a variant of this domain is reported to bind to PtdIns(3,4,5)P_3 _in vitro [29].

Although these domains are present in multiple organisms where they are involved in various cellular functions, their distribution across different species has not been studied. Most studies undertaken till date have been devoted towards understanding the function(s) of the individual proteins that contain these domains. Only a small number of studies have addressed the distribution of these proteins in a single species [30]. As these two domains are capable of binding to the same ligand we were curious to know if there was any selectivity for one over the other in different genomes. Towards this end we adopted a comparative genomics approach to study the distribution pattern of proteins containing these two domains across various eukaryotic lineages. Furthermore, to gain an understanding of the different cellular functions accomplished by such proteins, in different taxonomic groups, we analyzed the taxonomic distribution pattern of the additional domains that associate with these two domains. Our results reveal that although both FYVE and PX domains bind to the same ligand, PtdIns(3)P, there is a distinct selectivity for either of these two domains in individual genomes where both are present. Analysis of the domain architecture of these proteins indicates that while FYVE and PX domain proteins are involved in certain universal cellular functions, they have also been customized to accomplish group-specific functions by associating with certain group-specific domains.

## Results

### Distribution of FYVE and PX proteins in different taxonomic groups

**Figure 1:**
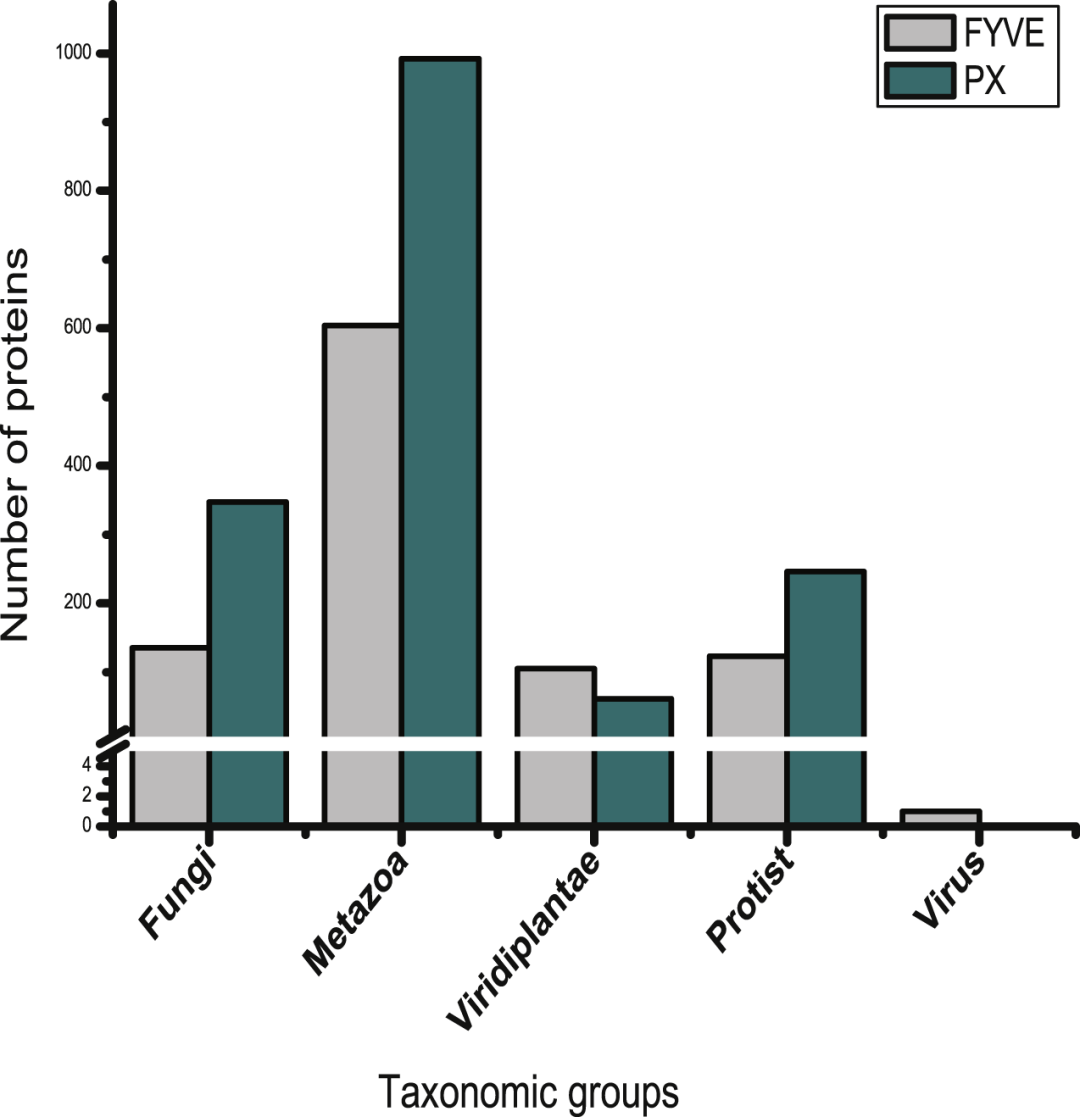
**Distribution of FYVE and PX domain proteins across different taxonomic groups**.

FYVE and PX domains bind to a common ligand, PtdIns(3)P [11]. In addition to PtdIns(3)P [22], PX also binds to PtdIns(3,4)P_2 _[23], PtdIns(4)P [24], PtdIns(4,5)P_2 _[25], PtdIns (3,5)P_2 _[26] and PtdIns(3,4,5)P_3 _[27] and the ligand-binding specificity of this domain is known to be dictated by the identity of residues at the ligand-binding site. By virtue of the greater versatility of its ligand-binding capability, it is expected that PX domain-containing proteins will perform many more functions than FYVE domain-containing proteins and there will be a greater number of the former in genomes compared to the latter. To test this hypothesis we have collected all the reported FYVE and PX domain containing sequences from NCBI protein database and have eliminated redundancy (see Methods). We analyzed the taxonomic classification of all these curated proteins. FYVE and PX domain proteins were found to be distributed amongst all four taxonomic groups of eukaryotes namely fungi, metazoa, viridiplantae and protist (Figure 1). Consistent with our hypothesis, higher occurrence of the PX domain compared to FYVE was observed in metazoa, fungi and protist. However, viridiplantae stands out as an exception as there is a higher occurrence of FYVE. In virus there is report of only one FYVE domain containing protein but no report of any PX domain protein as yet. The observed predominance of PX over FYVE proteins, in most taxonomic groups, may result from either all species, within a taxonomic group, having more PX than FYVE or if only a handful of species within the group encode an extremely large number of PX proteins in their genomes. To ascertain which of these two possibilities is correct we looked at the number of FYVE and PX proteins in genomes that are completely sequenced.

### Distribution of FYVE and PX proteins in individual genomes

**Figure 2:**
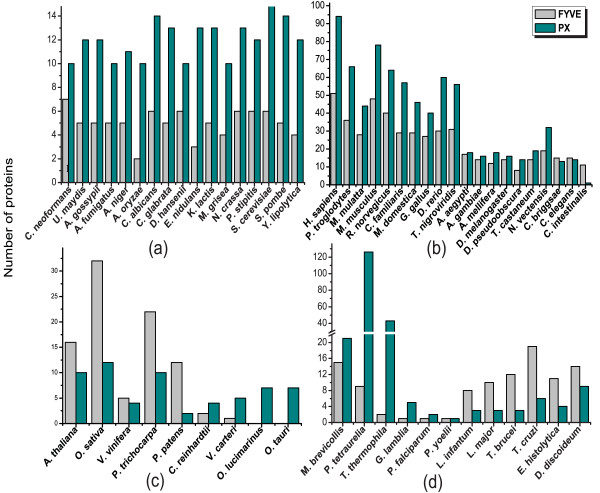
**Distribution of FYVE and PX domain proteins in completely sequenced genomes in different taxonomic groups**. Number of FYVE and PX proteins encoded by the genomes of (a) fungi, (b) metazoa, (c) viridiplantae and (d) protist.

To test if there is any distinct selectivity for either FYVE or PX at the individual genome level within a particular taxonomic group, we analyzed the abundance of FYVE and PX domain containing proteins of only those species (see Additional File 1) whose genomes have been completely sequenced (Figure 2). The observation that there are more PX than FYVE proteins in fungi (Figure 1) is also reflected at the species level as all of the seventeen completely-sequenced fungal genomes have more PX proteins than FYVE (Figure 2a). A similar trend, with a few exceptions, is discernible in completely sequenced metazoans as well (Figure 2b). The metazoan exceptions include *Caenorhabditis elegans, Caenorhabditis briggsae *and *Ciona intestinalis*. Like fungi and metazoa, in viridiplantae the relative abundance trend of the two domains observed for all available protein sequences is also maintained at the individual genome level, except in this case there appears to be a clear division in selectivity (for FYVE or PX) depending on whether the species belongs to the subphylum chlorophyta (green algae) or streptophyta (land plants and their relatives) (Figure 2c). The chlorophytes *Chlamydomonus reinhardtii *and *Volvox carteri *have more PX than FYVE proteins, while the chlorophytes *Ostreococcus lucimarinus *and *Ostreococcus tauri *do not encode any FYVE protein at all. Species belonging to streptophyta (*Arabidopsis thaliana, Oryza sativa, Vitis vinifera, Populus trichocarpa *and *Physcomitrella patens patens*) have more FYVE than PX proteins. In case of protist, there does not appear to be any overall selectivity for either FYVE or PX (Figure 2d) at the taxonomic level; some protist species have larger number of PX proteins than those with FYVE (*viz. Giardia lamblia, Paramecium tetraurelia, Tetrahymena thermophila, Monosiga brevicollis*, and *Plasmodium falciparum*) while a comparable number of species have more FYVE proteins than PX (*viz. Leishmania major, Leishmania infantum, Trypanosoma brucei, Trypanosoma cruzi, Entamoeba histolytica*, and *Dictyostelium discoideum*). Interestingly in *Plasmodium yoelii *the two types of proteins are present in equal number. Thus the protist taxonomic group contains an almost equal number of species with either an excess of FYVE or an excess of PX. Prior phylogenetic studies reveal that unlike the fungi, metazoa and viridiplantae taxonomic groups, the lineage of the protist group is unclear as there is ambiguity regarding when the main branches of the present day protist species diverged from each other [31]. In fact protists as a group are paraphyletic as some members of this group are closer to non-protists than to other protists and this may explain the observed heterogeneity in the distribution pattern of FYVE and PX in this group. The lack of predominance of species with more PX than FYVE in their genomes, within the protist taxonomic group, is in contradiction of the observed overall excess of PX proteins in protists (Figure 1). However this may be because of a small number of genomes encoding an unusually large number of PX proteins. In concurrence with this, significantly higher number of PX domain has been detected in at least two species, *Paramecium tetraurelia *and *Tetrahymena thermophila*, (Figure 2d- broken bars). Therefore, with the exception of protists, by and large the trend observed for all available protein sequences is also maintained at the individual genome level and is indicative of selectivity for either FYVE (in streptophyta of viridiplantae) or PX (fungi and metazoa) domain.

### Distribution of associated domains

**Figure 3:**
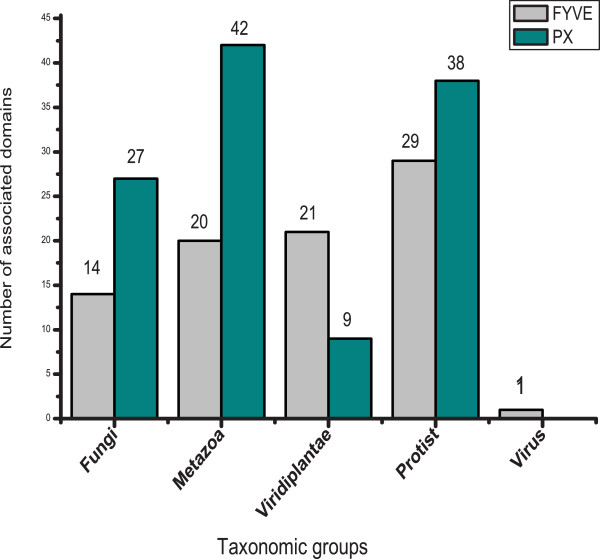
**Number of associated domains of FYVE and PX in different lineages**.

There appears to be selectivity for either FYVE or PX domain in most taxonomic groups. Given that both domains have the potential to bind the same ligand, PtdIns(3)P [4,11], the domain specifically selected in a given taxonomic group is expected to be involved in more PtdIns(3)P dependent cellular functions and thus will be associated with a greater variety of other domains to discharge these functions. Therefore, while PX proteins are expected to have greater diversity in terms of domain architecture in metazoa and fungi, FYVE proteins are expected to associate with a greater variety of domains in viridiplantae. To test this hypothesis we have analyzed the domains that associate with FYVE and PX. Our result shows that 63% of FYVE domain-containing proteins and 52% of PX domain-containing proteins associate with at least one other domain listed in Pfam-A database (data not shown). Of the 10340 domains listed in Pfam-A database, 58 and 85 domains were assigned to be extant in proteins with FYVE and PX domains respectively. Figure 3 summarizes the number of domains associating with FYVE and PX proteins in the different taxonomic groups. Once again a direct correlation is evident between the relative abundance trend of FYVE vs. PX in a given taxonomic group and the number of domains that associate with them. For example, in fungi and metazoa, which have higher abundance of PX compared to FYVE, there are more domains associating with the former compared to the latter. The same correlation is observed in the case of viridiplantae; more domains are associated with FYVE, which is more abundant compared to PX in this taxonomic group. In all three cases the number of domains associating with the more abundant domain is almost twice the number of domains that associate with the less abundant one (Figure 3, see Fungi, Metazoa and Viridiplantae). In case of protist, a taxonomic group with no apparent selectivity for either FYVE or PX, although a greater number of domains are found to associate with PX, the number of domains associating with FYVE is also significant (29 for FYVE as opposed to 38 for PX) (Figure 3). Once again the slightly larger number of domains associating with PX could be because of the plethora of such proteins in *Paramecium tetraurelia *and *Tetrahymena thermophila *genomes (Figure 2d). Thus higher number of domains is found to associate with the PtdIns(3)P-binding domain that is more abundant in a given taxonomic group indicating that a greater number of PtdIns(3)P-dependent functions are discharged using that particular domain.

**Figure 4:**
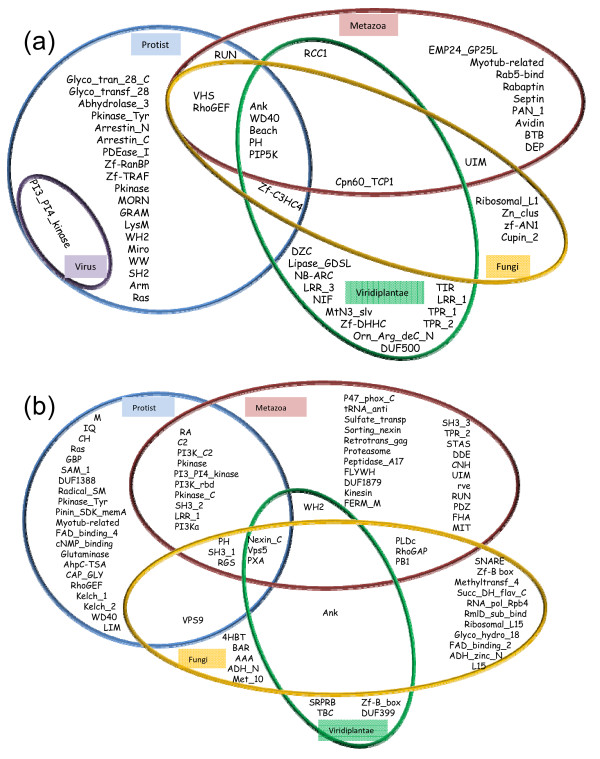
**Distribution of associated domains across different taxonomic groups**. Venn diagram of the domains associated with (a) FYVE and (b) PX proteins.

Analysis of the taxonomic distribution of the domains associating with FYVE and PX (Figure 4a and 4b) shows that five domains are found to associate with FYVE in all taxonomic groups (Ank, WD40, Beach, PH and PIP5K) while three domains are common amongst all taxonomic groups with respect to PX (Nexin_C, Vps5 and PXA). Most domains appear to be taxonomic group specific as 78% of FYVE and 74% of PX associated domains are present exclusively in a unique taxonomic group (Figure 4a &4b). The taxonomic distribution of domains shows an interesting trend. Depending on whether FYVE or PX is more abundant in a given taxonomic group, the number of domains exclusively associating with it (FYVE/PX) is larger. For example in metazoa, whose genome encodes more PX proteins, 22 domains that are found to co-occur do not associate with PX in any other taxonomic group. On the other hand, only 9 domains associate exclusively with metazoan FYVE proteins. In fungi, another group with selectivity for PX, the corresponding numbers are 16 and 4 for PX and FYVE domain proteins respectively. The reverse is observed for viridiplantae, a taxonomic group with more FYVE compared to PX. While 13 domains associate exclusively with FYVE proteins, only 4 domains associate exclusively with viridiplantae PX proteins. Consistent with a lack of selectivity for either FYVE or PX in protist, almost equal number of domains associate exclusively with these two domains in this taxonomic group (19 for FYVE and 21 for PX). The results indicate that while some associating domains are distributed across multiple taxonomic groups, most are group specific.

**Figure 5:**
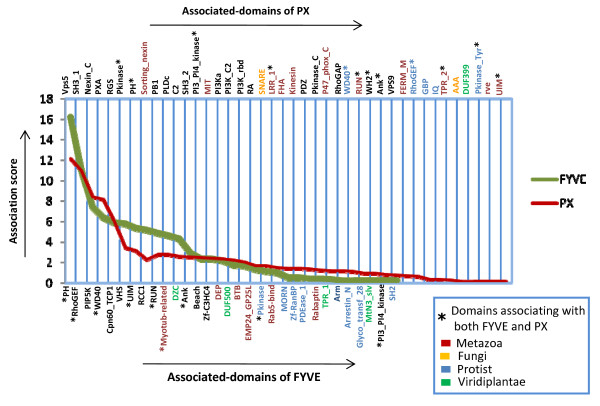
**Association score distribution graph of FYVE and PX associated domains**. The associated domains of FYVE and PX proteins are plotted according to their association score. The upper horizontal axis shows the associated domains of PX and its corresponding graph is drawn in red. The lower horizontal axis is for the associated domains of FYVE and the corresponding graph is drawn in green. Domain names in black font are present in more than one taxonomic group whereas domains that are found only in a particular taxonomic group are coloured according to the colour code given in the figure. Associated domains which have just a single representative have not been included in this graph. They are included in the graph given in Additional File 4. Domains marked with * are associated with both FYVE and PX. Although LRR_1, TPR_2, Pkinase_Tyr in the PX axis and Myotub-related in the FYVE axis are marked with *, they are absent in one of the axes as they are represented only once in the corresponding dataset.

The associated domains that are found in multiple taxonomic groups are expected to be more prevalent in the FYVE and PX protein repertoire compared to those that are group specific. To test this hypothesis we have calculated the association score of all the domains (see Methods and tables in Additional File 2 &3) and plotted them for both FYVE and PX (Figure 5). In both cases domains such as PH in case of FYVE and Vps5 in case of PX, that are extant in all taxonomic groups have the highest association score in their respective dataset and these domains are at least 3.5 times more prevalent compared to the highest scoring domain that exclusively occurs in only one taxonomic group (Myotub_related for FYVE and Sorting_nexin in PX) (Figure 5). In fact the five domains in each set that have the highest association score are present in at least three out of four taxonomic groups (Figure 4 and 5). The graph also reveals that most group-specific domains have low association frequencies (also see Additional File 4). These observations indicate that certain FYVE and PX domain-dependent functions are likely to have emerged early in the evolutionary process as selective domain combinations are present in all taxonomic groups and also the high association frequencies of these combinations suggest that they have been retained even through prolonged evolutionary changes of eukaryotic lineages. Also, the group specific rare domain architectures might have emerged to accomplish certain group-specific functions. It is worth noting that some domains are found to associate with both FYVE and PX and these are marked with asterix in Figure 5. Of these most have a higher propensity of co-occurring with FYVE (PH, RhoGEF, WD40, RUN, Myotub-related, Ank, and UIM), while some show selectivity for PX (Pkinase, PI3_PI4_kinase and LRR_1).

### Functional categorization of associated domains

The large variety of domains associating with FYVE and PX indicates that diverse cellular functions are discharged by proteins containing these lipid-binding modules. In this study we have functionally categorized the associating domains following the functional annotations of Pfam (Table 1). Although the associated domains are involved in assorted cellular activities, there is a distinct selectivity for utilizing the FYVE and PX proteins in only a small subset of these functions. The 10 most abundant domains associating with FYVE are involved in cellular processes such as signal transduction, intracellular trafficking, cell division and chaperone activity, while the comparable domains co-occurring with PX show involvement in signal transduction, intracellular trafficking and cytoskeletal regulation. Involvement of these proteins in cellular processes such as inorganic ion transport, defence mechanisms, transcriptional and translational regulation is minimal. For example Ribosomal_L1 and Zn_clus domains that are involved in translation and transcription respectively, have a very low association score with FYVE. Therefore, there appears to be selectivity for utilizing the FYVE and PX domain-containing proteins for certain types of cellular functions and involvement of such proteins in other functions is not very significant.

**Table 1:** Functional classification of domains associated with FYVE and PX

Functional categories	FYVE-associated domains	PX-associated domains
Signal transduction mechanisms	PH, RhoGEF, PIP5K, RUN, DEP, PDEase_I, Arm, Arrestin_N, SH2, Arrestin_C, Miro, NB-ARC, Ras, TIR, zf-TRAF	PH, RhoGEF, RUN, Ras, RGS, PB1, C2, PI3K_C2, RA, FHA, PDZ, Pkinase_C, RhoGAP, IQ, CH

Intracellular trafficking, secretion, and vesicular transport	VHS, EMP24_GP25L, Rab5-bind, Zf-RanBP, Rabaptin	Vps5, Nexin_C, Sorting_nexin, MIT, PI3Ka, SNARE, Kinesin, VPS9, BAR, SRPRB, TBC

Chaperone	Cpn60_TCP1	AAA

Cell cycle control, cell division, chromosome partitioning	RCC1, DZC, Septin	PI3K_rbd

Cytoskeleton	PH, WH2	PH, WH2, SH3_1, SH3_2, SH3_3, FERM_M, CAP_GLY, CH

Transcription	Zn_clus	

Translation	Ribosomal_L1	L15, Ribosomal_L15, tRNA_anti

Defence mechanisms		p47_phox_C, GBP

Inorganic ion transport		Sulfate_transp

Protein-protein interaction	WD40, UIM, Ank, zf-C3HC4, BTB, TPR_1, LRR_1, LRR_3, PAN_1, WW, zf_AN1	WD40, UIM, Ank, LRR_1

Catalytic	Pkinase, Glyco_transf_28, PI3_PI4_kinase, Abhydrolase_3, Glyco_tran_28_C, Lipase_GDSL, Orn_Arg_deC_N, Pkinase_Tyr, Myotub-related	Pkinase, PI3_PI4_kinase, Pkinase_Tyr, PLDc, 4HBT, ADH_N, ADH_zinc_N, AhpC-TSA, rve, glutaminase, Glyco_hydro_18, Myotub-related, Proteasome, Radical_SAM, RmlD_sub_bind, RNA_pol_Rpb4

Metabolism		FAD_binding_2, FAD_binding_4, Succ_DH_flav_C

Replication, recombination & repair		Retrotrans_gag

General function prediction only	GRAM, LysM	cNMP_binding, DDE, FLYWCH, Methyltransf_4, Peptidase_A17, Zf-B_box

Function unclear	Beach, DUF500, MORN, MtN3_slv, Cupin_2, NIF, TPR_2, zf-DHHC	TPR_2, PXA, DUF399, CNH, DUF1388, DUF1879, Kelch_1, Kelch_2, LIM, M, Met_10, Pinin_SDK_memA, SAM_1, STAS, UPF0047

The functional categories of the associated domains were adopted from the COG classification of proteins. Functions were assigned to the domains following Pfam annotations.

### Specialization of FYVE domain in viridiplantae

**Figure 6:**
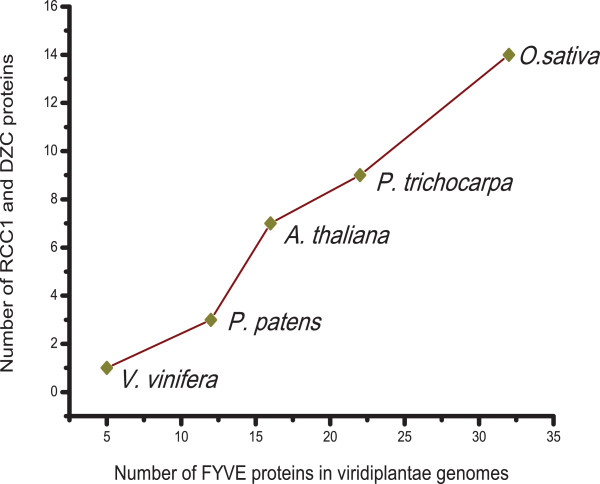
**Correlation between number of proteins with FYVE-RCC1-DZC and number of FYVE proteins in viridiplantae genomes**. The number of FYVE proteins that associate with RCC1 and DZC are plotted against the total number of FYVE proteins in genomes of streptophytes that encode higher number of FYVE proteins compared to PX.

**Figure 7:**
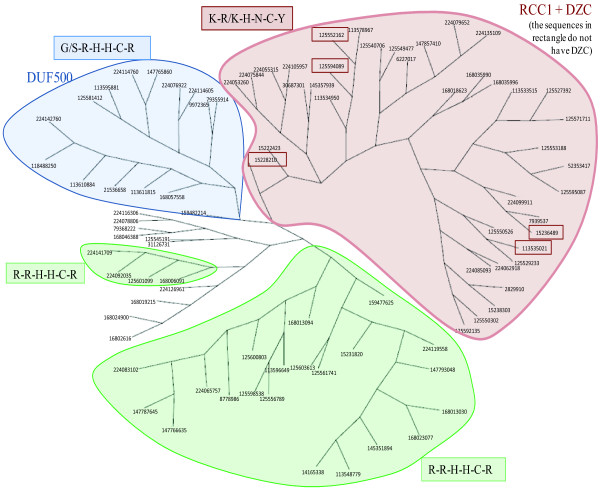
**Unrooted phylogenetic tree of viridiplantae FYVE domains**. Tree was reconstructed using protein parsimony method of PHYLIP program using the amino acid sequence of the entire FYVE domain. The coloured zones of the tree show clustering on the basis of variation in the consensus sequence at the PI-binding site. FYVE domains in the green cluster have the canonical ligand-binding site with the consensus of R-R-H-H-C-R. The red cluster shows a non-canonical consensus of K-R/K-H-N-C-Y and these FYVE proteins associate with RCC1 and DZC domains, while the blue cluster has the non-canonical consensus of G/S-R-H-H-C-R and associates with DUF500.

We have already shown that most of the members of the taxonomic group viridiplantae exhibit the selective use of FYVE proteins (Figure 1 and Figure 2c). We analyzed the domain architecture of FYVE proteins specifically in this taxonomic group. While a great variety of domains do associate with FYVE, DZC is the most abundant viridiplantae-specific domain (Figure 5). Proteins that contain FYVE and DZC domain also always contain the RCC1 domain. In fact the association of RCC1 with FYVE can be considered plant-specific, as there is only a single instance of a RCC1 co-occurring with FYVE outside the viridiplantae taxonomic group, namely in the metazoa *N. vectensis*. Figure 6 shows that there is a direct correlation between the number of proteins of this specific architecture (with FYVE, RCC1 and DZC) and the prevalence of FYVE proteins in individual streptophyte genomes. The high prevalence of proteins with this architecture and their direct correlation with number of FYVE proteins indicates that this unique domain combination is likely to have emerged to accomplish plant-specific function(s). We were curious to know if the FYVE domains present in these plant-specific proteins had evolved together to display any novel characteristics. Towards this end we reconstructed a phylogenetic tree on the basis of entire FYVE domain from all FYVE protein sequences from viridiplantae and observed clustering of domains with signature ligand-binding site consensus patterns (Figure 7). While one of the clusters has the canonical ligand-binding site consensus sequence of R-R-H-H-C-R, two clusters display deviation. The first of the two deviant clusters has FYVE-RCC1-DZC architecture with a ligand-binding site sequence of K-R/K-H-N-C-Y. Such a non-canonical FYVE domain from *A. thaliana*, has been shown to bind to PtdIns(3,4,5)P_3 _with better affinity than PtdIns(3)P in vitro [29]. However, given that there is a general consensus regarding the lack of PtdIns(3,4,5)P_3 _in plants [32], the functional significance of such an in vitro binding remains debatable [33]. The second non-canonical FYVE domain, with a ligand-binding site sequence of G/S-R-H-H-C-R, associates with the plant-specific domain DUF500 (Figure 4a). Therefore there appears to be a correlation between ligand-binding site sequence of FYVE domains and the domain architecture of these proteins. Also the deviants actually constitute a larger number of plant FYVE proteins rather than those with canonical ligand-binding sites. The above observations indicate that in course of evolution, the lipid signaling system of higher plants have not only selectively favoured the use of FYVE, rather than PX in their cellular processes; but have also adopted unique domain architectures supported by special modification around lipid binding site in order to better accomplish these processes.

## Discussion

We have adopted a comparative genomics approach in order to extend our understanding of PtdIns(3)P-mediated cellular functions. PtdIns(3)P is recognized mainly by two cellular domains, FYVE and PX [4]. We have analyzed the number of FYVE and PX proteins in different taxonomic groups as well as in individual completely sequenced genomes. Our study has revealed that most taxonomic groups show selectivity for either of the two. We have also observed a correlation between the selectively utilized domain and the diversity of domains associating with it, indicating that the more abundant domain is used to accomplish a greater variety of group-specific functions (Figure 3 and Figure 5). Interestingly, some domains are extant in both FYVE and PX proteins (Figure 5). However in such cases there is a clear selectivity of co-occurrence with either FYVE or PX. For example while RhoGEF and UIM have high association frequencies in the FYVE dataset, they rarely co-occur with PX. The reverse is true for Pkinase and PI3_PI4_kinase which selectively associate with PX. The only exception is the PH domain, which has high association frequencies in both the datasets. This is not surprising because the PH domain is found to be highly prevalent in genomes and is termed as 'promiscuous' for its ability to associate with a very large number of other domains [34].

Our results show that most genomes have a higher number of PX proteins. This may be indicative of this domain being selectively utilized for PtdIns(3)P specific functions. However, given that PX domain is documented to bind ligands other than PtdIns(3)P [22-27], it could also indicate that PX functions that are independent of PtdIns(3)P may play more significant roles in these organisms. Interestingly, there are two completely sequenced genomes which do not encode any FYVE protein (*O. lucimarinus *and *O. tauri *in Figure 2c) but of the 58 fully-sequenced genomes that have been analyzed (Additional File 1) there is not a single instance of any genome that does not encode PX proteins, indicating that the latter domain may be indispensible for cellular functions.

PtdIns(3)P is a minor constituent of cellular membranes [35] and most of it is confined to endosomal surface [7]. Thus if all the FYVE and PX domains in a given genome do bind to PtdIns(3)P, then organisms whose genomes encode a high number of such proteins may have higher amounts of PtdIns(3)P compared to those which encode fewer PtdIns(3)P binding proteins. Alternatively it is possible that the expression of such proteins are spatially and/or temporally separated, with only a subset of them being expressed simultaneously within a given cell type at a certain time. The latter scenario is more plausible because in most cases the combined number of FYVE and PX proteins increases with the increase in organismal complexity. For example while *S. cerevisiae *and *S. pombe*, both unicellular eukaryotes, have around 20 proteins capable of binding PtdIns(3)P, the number of such proteins is well over 100 for highly evolved multicellular species such as *H. sapiens, P. troglodytes, M. musculus *etc.

One of the intriguing observations of our study is that streptohytes have higher number of FYVE proteins compared to PX. This excess of FYVE may be attributed to the presence of FYVE protein(s) that are involved only in plant-specific functions. One such plant-specific activity is the formation of cell plate during cytokinesis in which vesicular trafficking plays an important role. Therefore, cell division in plants may require proteins that can function in vesicular trafficking as well as chromosome segregation. Our results show that higher number of FYVE proteins in streptophytes is most likely due to amplification of genes encoding proteins that contain RCC1, DZC and FYVE domains (Figure 6). Interestingly, RCC1 is found to associate with chromosomes and has a well documented role in cell division [36]. Thus this family of proteins has the potential to function both in chromosome segregation (RCC1) as well as vesicular trafficking (FYVE). Proteins with this particular domain architecture is completely absent in chlorophytes (green algae), which is an early diverging class within the green plant lineage [31]. While higher order green algae such as *Chlamydomonas reinhardtii *and *Volvox carteri*, do encode FYVE proteins, the number of such proteins is lower than that of PX proteins encoded by these genomes. In addition none of these FYVE domains co-occur with either RCC1 or DZC. In fact lower order green algae such as *Ostreococcus tauri *and *Ostreococcus lucimarinus *do not encode any FYVE proteins at all. This complete absence of FYVE proteins in the genomes of *Ostreococcus *sp. is not unusual as these are characterized by minimal cellular organization with a well documented absence of several proteins that are present in higher plants [37]. Therefore the greater abundance of FYVE proteins in higher plants may be resulting from a divergence event between chlorophytes (green algae) and streptophytes (land plants). In metazoa a similar deviation from the trend displayed by a majority is observed in nematodes (*C. elegans *and *C. briggsae*) and ascidia (*C. intestinalis*) as these have more FYVE proteins compared to PX. However, further studies are necessary to ascertain the reason for this deviation.

## Conclusions

In this study we have analysed the distribution of FYVE and PX proteins in different taxonomic groups. There is a distinct selectivity for either FYVE or PX in individual genomes where both are present. While fungi and metazoa have higher number of PX, streptophyta of viridiplantae have a higher number of FYVE. Presence of proteins with FYVE, RCC1 and DZC domain combination, in the genomes of streptophytes, may be the likely explanation for more FYVE proteins in these viridiplantae species. We have also analyzed the taxonomic distribution of domains co-occurring with FYVE or PX and observed that depending on whether FYVE or PX is more abundant in a given taxonomic group, the number of domains exclusively associating with it (FYVE/PX) in that particular taxonomic group is larger. This result indicates that the more abundant domain may be involved in a greater number of cellular functions. Thus, our study of the taxonomic distribution of FYVE and PX domains, as well as the domains co-occurring with these, provides insights into the architectural and functional diversity of these proteins. This study demonstrates the importance of comparative genomics approach for gaining a holistic understanding of protein families.

## Methods

### Data collection

Protein sequences containing FYVE and PX domains were collected separately from NCBI protein database (http://www.ncbi.nlm.nih.gov) [38] using keyword search facility of Entrez. Redundancy from both the sequence sets was removed by clustering the sequences at 95% identity level, using the CD-HIT program [39-41] version 2007-0103. The clusters were next screened to prevent the elimination of inter-species identical proteins. For a list of completely sequenced organisms that were used in this analysis please see Additional file 1.

### Domain architecture and taxonomic classification of the sequences

Domains were assigned to the protein sequences based on the domains stored in Pfam-A section of the Pfam database release 22.0 (having 9318 families) [42], using the HMMER-2.3.2 program [43] with an E-value cut-off of 0.1. Based on NCBI taxonomy database [38,44], the FYVE and PX protein sequences were classified into 5 major taxonomic groups namely metazoa, fungi, viridiplantae, protist and virus.

### Functional classification of the associated domains

The functional categories of the associated domains that were adopted from the COG classification of proteins ftp://ftp.ncbi.nih.gov/pub/COG/COG/fun.txt[45] include: (i) signal transduction mechanisms (ii) intracellular trafficking, secretion, and vesicular transport (iii) chaperones (iv) cell cycle control, cell division, chromosome partitioning (v) cytoskeleton (vi) transcription (vii) translation (viii) defence mechanisms (ix) inorganic ion transport (x) metabolism (xi) replication, recombination and repair (xii) general function prediction only and (xiii) function unknown. Two more categories namely (xiv) protein-protein interaction and (xv) catalytic were further incorporated in the list. Functions were assigned to the domains following Pfam annotations.

### Calculation of association score of domains

Association score of each domain type, r, associating with FYVE/PX proteins was calculated as per the following formula

where A_r _is the number of FYVE/PX proteins containing the associated domain type r and N is the summation of the total number of each associated domain type found in FYVE/PX proteins.

### Phylogenetic analysis

Multiple sequence alignment (MSA) was carried out with the amino acid sequences of the entire FYVE domains belonging to the viridiplantae taxonomic group using ClustalW [46]. The MSA was then used for unweighted maximum-parsimony phylogenetic reconstruction using the Protpars program of PHYLIP v. 3.67 [47]. The resultant unrooted tree was drawn using Drawtree of PHYLIP.

## Competing interests

The authors declare that they have no competing interests.

## Authors' contributions

SB^1 ^acquired the data, designed and performed the experiments, analyzed and interpreted the results, prepared the figures and tables, and helped in drafting the manuscript. SB^2 ^supervised data acquisition and processing, designed the experiments, analyzed and interpreted the results and helped in drafting the manuscript. SS conceived of the study, designed the experiments, analyzed and interpreted the results and drafted the manuscript. All the authors have read and approved the final manuscript.

## Supplementary Material

Additional file 1 **List of completely sequenced organisms used in this analysis**. This file enlists the names of the completely sequenced organisms that are included in the current analysis. Click here for file

Additional file 2 **Domains associating with FYVE proteins**. List of all the domains that are found to associate with the downloaded FYVE proteins. Their distribution in various taxonomic groups is also included. Click here for file

Additional file 3 **Domains associating with PX proteins**. List of all the domains that are found to associate with the downloaded PX proteins. Their distribution in various taxonomic groups is also included. Click here for file

Additional file 4 **Association score distribution graph of all FYVE and PX associated domains**. The associated domains of FYVE and PX proteins are plotted according to their association score. The upper horizontal axis shows the associated domains of PX and its corresponding graph is drawn in red. The lower horizontal axis is for the associated domains of FYVE and the corresponding graph is drawn in green. Domain names in black font are present in more than one taxonomic group whereas domains that are found only in a particular taxonomic group are coloured according to the colour code given in the figure. Click here for file
