# Supplementary material for: Comparative genomics reveals selective distribution and domain organization of FYVE and PX domain proteins across eukaryotic lineages
Source: BMC Genomics. 2010 Feb 2;11:83. doi: 10.1186/1471-2164-11-83 (PMC2837644; doi:10.1186/1471-2164-11-83)
Supplement: Additional file 1 — List of completely sequenced organisms used in this analysis. This file enlists the names of the completely sequenced organisms that are included in the current analysis. [file 1471-2164-11-83-S1.PDF]

<u>**List of completely sequenced organisms**</u>

Aedes aegypti

Anopheles gambiae

Apis mellifera

Arabidopsis thaliana

Ashbya gossypii

Aspergillus fumigatus

Aspergillus niger

Aspergillus oryzae

Caenorhabditis briggsae

Caenorhabditis elegans

Candida albicans

Candida glabrata

Canis familiaris

Chlamydomonas reinhardtii

Ciona intestinalis

Cryptococcus neoformans

Danio rerio

Debaromyces hansenii

Dictyostelium discoideum

Drosophila melanogaster

Drosophila pseudoobscura

Emericella nidulans

Entamoeba histolytica

Gallus gallus

Giardia lamblia

Homo sapiens

Kluyveromyces lactis

Leishmania infantum

Leishmania major

Macaca mulatta

Magnaporthe grisea

Monodelphis domestica

Monosiga brevicollis

Mus musculus

Nematostella vectensis

Neurospora crassa

Oryza sativa

Ostreococcus lucimarinus

Ostreococcus tauri

Pan troglodytes

Paramecium tetraurelia

Physcomitrella patens patens

Pichia stipitis

Plasmodium falciparum

Plasmodium yoelii

Populus trichocarpa

Rattus norvegicus

Saccharomyces cerevisiae

Schizosaccharomyces pombe

Tetrahymena thermophila

Tetraodon nigroviridis

Tribolium castaneum

Trypanosoma brucei

Trypanosoma cruzi

Ustilago maydis

Vitis vinifera

Volvox carteri

Yarrowia lipolytica
