# Supplementary material for: Comparative genomics reveals selective distribution and domain organization of FYVE and PX domain proteins across eukaryotic lineages
Source: BMC Genomics. 2010 Feb 2;11:83. doi: 10.1186/1471-2164-11-83 (PMC2837644; doi:10.1186/1471-2164-11-83)
Supplement: Additional file 2 — Domains associating with FYVE proteins. List of all the domains that are found to associate with the downloaded FYVE proteins. Their distribution in various taxonomic groups is also included. [file 1471-2164-11-83-S2.PDF]

Associated domains of FYVE

Serial number	Associated domain	Association score	Total	Metazoa	Fungi	Viridiplantae	Protist	Virus
1	PH	16.20	157	132	1	3	21	-
2	RhoGEF	11.04	107	90	1	-	16	-
3	PIP5K	7.43	72	25	26	12	9	-
4	WD40	6.40	62	57	1	2	2	-
5	Cpn60_TCP1	5.88	57	22	24	11	-	-
6	VHS	5.78	56	29	26	-	1	-
7	UIM	5.37	52	26	26	-	-	-
8	RCC1	5.16	50	1	-	49	-	-
9	RUN	4.85	47	46	-	-	1	-
10	Myotub-related	4.64	45	45	-	-	-	-
11	DZC	4.33	42	-	-	42	-	-
12	Ank	2.89	28	21	2	4	1	-
13	Beach	2.37	23	19	1	2	1	-
14	zf-C3HC4	2.37	23	-	21	1	1	-
15	DEP	2.17	21	21	-	-	-	-
16	DUF500	1.75	17	-	-	17	-	-
17	BTB	1.65	16	16	-	-	-	-
18	EMP24_GP25L	1.34	13	13	-	-	-	-
19	Pkinase	1.13	11	-	-	-	11	-
20	Rab5-bind	1.03	10	10	-	-	-	-
21	MORN	0.51	5	-	-	-	5	-
22	zf-RanBP	0.51	5	-	-	-	5	-
23	PDEase_I	0.41	4	-	-	-	4	-
24	Rabaptin	0.41	4	4	-	-	-	-
25	TPR_1	0.31	3	-	-	3	-	-
26	Arm	0.21	2	-	-	-	2	-
27	Arrestin_N	0.21	2	-	-	-	2	-
28	Glyco_transf_28	0.21	2	-	-	-	2	-
29	MtN3_slv	0.21	2	-	-	2	-	-
30	PI3_PI4_kinase	0.21	2	-	-	-	1	1
31	SH2	0.21	2	-	-	-	2	-
32	Abhydrolase_3	0.10	1	-	-	-	1	-
33	Arrestin_C	0.10	1	-	-	-	1	-
34	Avidin	0.10	1	1	-	-	-	-
35	Cupin_2	0.10	1	-	1	-	-	-
36	Glyco_tran_28_C	0.10	1	-	-	-	1	-
37	GRAM	0.10	1	-	-	-	1	-
38	Lipase_GDSL	0.10	1	-	-	1	-	-
39	LRR_1	0.10	1	-	-	1	-	-
40	LRR_3	0.10	1	-	-	1	-	-
41	LysM	0.10	1	-	-	-	1	-
42	Miro	0.10	1	-	-	-	1	-
43	NB-ARC	0.10	1	-	-	1	-	-
44	NIF	0.10	1	-	-	1	-	-
45	Orn_Arg_deC_N	0.10	1	-	-	1	-	-
46	PAN_1	0.10	1	1	-	-	-	-
47	Pkinase_Tyr	0.10	1	-	-	-	1	-
48	Ras	0.10	1	-	-	-	1	-
49	Ribosomal_L1	0.10	1	-	1	-	-	-
50	Septin	0.10	1	1	-	-	-	-
51	TIR	0.10	1	-	-	1	-	-
52	TPR_2	0.10	1	-	-	1	-	-
53	WH2	0.10	1	-	-	-	1	-
54	WW	0.10	1	-	-	-	1	-
55	zf-AN1	0.10	1	-	1	-	-	-
56	zf-DHHC	0.10	1	-	-	1	-	-
57	zf-TRAF	0.10	1	-	-	-	1	-
58	Zn_clus	0.10	1	-	1	-	-	-
