# Supplementary material for: Comparative genomics reveals selective distribution and domain organization of FYVE and PX domain proteins across eukaryotic lineages
Source: BMC Genomics. 2010 Feb 2;11:83. doi: 10.1186/1471-2164-11-83 (PMC2837644; doi:10.1186/1471-2164-11-83)
Supplement: Additional file 3 — Domains associating with PX proteins. List of all the domains that are found to associate with the downloaded PX proteins. Their distribution in various taxonomic groups is also included. [file 1471-2164-11-83-S3.PDF]

Associated domains of PX

Serial number	Associated domain	Association score	Total	Metazoa	Fungi	Viridiplantae	Protist
1	Vps5	12.12	186	115	39	23	9
2	SH3_1	10.94	168	128	35	-	5
3	Nexin_C	8.40	129	80	37	11	1
4	PXA	8.14	125	76	36	11	2
5	RGS	6.06	93	66	25	-	2
6	Pkinase	3.45	53	44	-	-	9
7	PH	3.13	48	30	15	-	3
8	Sorting_nexin	2.3	46	46	-	-	-
9	PB1	2.74	42	19	23	-	-
10	PLDc	2.74	42	40	2	-	-
11	C2	2.6	40	38	-	-	2
12	SH3_2	2.54	39	37	-	-	2
13	PI3_PI4_kinase	2.47	38	37	-	-	1
14	MIT	2.41	37	37	-	-	-
15	PI3Ka	2.34	36	35	-	-	1
16	PI3K_C2	2.21	34	33	-	-	1
17	PI3K_rbd	2.02	31	30	-	-	1
18	RA	1.63	25	24	-	-	1
19	SNARE	1.63	25	-	25	-	-
20	LRR_1	1.50	23	22	-	-	1
21	FHA	1.43	22	22	-	-	-
22	Kinesin	1.43	22	22	-	-	-
23	PDZ	1.37	21	21	-	-	-
24	Pkinase_C	1.24	19	18	-	-	1
25	p47_phox_C	1.17	18	18	-	-	-
26	RhoGAP	1.17	18	3	15	-	-
27	WD40	1.11	17	-	-	-	17
28	RUN	0.91	14	14	-	-	-
29	WH2	0.91	14	13	-	1	-
30	Ank	0.78	12	-	10	2	-
31	VPS9	0.72	11	-	10	-	1
32	FERM_M	0.65	10	10	-	-	-
33	RhoGEF	0.59	9	-	-	-	9
34	GBP	0.32	5	-	-	-	5
35	IQ	0.33	5	-	-	-	5
36	TPR_2	0.26	4	4	-	-	-
37	AAA	0.13	2	-	2	-	-
38	DUF399	0.13	2	-	-	2	-
39	Pkinase_Tyr	0.13	2	-	-	-	2
40	rve	0.13	2	2	-	-	-
41	UIM	0.13	2	2	-	-	-
42	4HBT	0.06	1	-	1	-	-
43	ADH_N	0.06	1	-	1	-	-
44	ADH_zinc_N	0.06	1	-	1	-	-
45	AhpC-TSA	0.06	1	-	-	-	1
46	BAR	0.06	1	-	1	-	-
47	CAP_GLY	0.06	1	-	-	-	1
48	CH	0.06	1	-	-	-	1
49	CNH	0.06	1	1	-	-	-
50	cNMP_binding	0.06	1	-	-	-	1
51	DDE	0.06	1	1	-	-	-
52	DUF1388	0.06	1	-	-	-	1
53	DUF1879	0.06	1	1	-	-	-
54	FAD_binding_2	0.06	1	-	1	-	-
55	FAD_binding_4	0.06	1	-	-	-	1
56	FLYWCH	0.06	1	1	-	-	-
57	Glutaminase	0.06	1	-	-	-	1
58	Glyco_hydro_18	0.06	1	-	1	-	-
59	Kelch_1	0.06	1	-	-	-	1
60	Kelch_2	0.06	1	-	-	-	1
61	L15	0.06	1	-	1	-	-
61	LIM	0.06	1	-	-	-	1
63	M	0.06	1	-	-	-	1
64	Met_10	0.06	1	-	1	-	-
65	Methyltransf_4	0.06	1	-	1	-	-
66	Myotub-related	0.06	1	-	-	-	1
67	Peptidase_A17	0.06	1	1	-	-	-
68	Pinin_SDK_memA	0.06	1	-	-	-	1
69	Proteasome	0.06	1	1	-	-	-
70	Radical_SAM	0.06	1	-	-	-	1
71	Ras	0.06	1	-	-	-	1
72	Retrotrans_gag	0.06	1	1	-	-	-
73	Ribosomal_L15	0.06	1	-	1	-	-
74	RmlD_sub_bind	0.06	1	-	1	-	-
75	RNA_pol_Rpb4	0.06	1	-	1	-	--
76	SAM_1	0.06	1	-	-	-	1
77	SH3_3	0.06	1	1	-	-	-
78	SRPRB	0.06	1	-	-	1	-
79	STAS	0.06	1	1	-	-	-
80	Succ_DH_flav_C	0.06	1	-	1	-	-
81	Sulfate_transp	0.06	1	1	-	-	-
82	TBC	0.06	1	-	-	1	-
83	tRNA_anti	0.06	1	1	-	-	-
84	UPF0047	0.06	1	-	1	-	-
85	zf-B_box	0.06	1	-	-	1	-
