# Supplementary figures and images for: Comparative genomics reveals selective distribution and domain organization of FYVE and PX domain proteins across eukaryotic lineages
Source: BMC Genomics. 2010 Feb 2;11:83. doi: 10.1186/1471-2164-11-83 (PMC2837644; doi:10.1186/1471-2164-11-83)

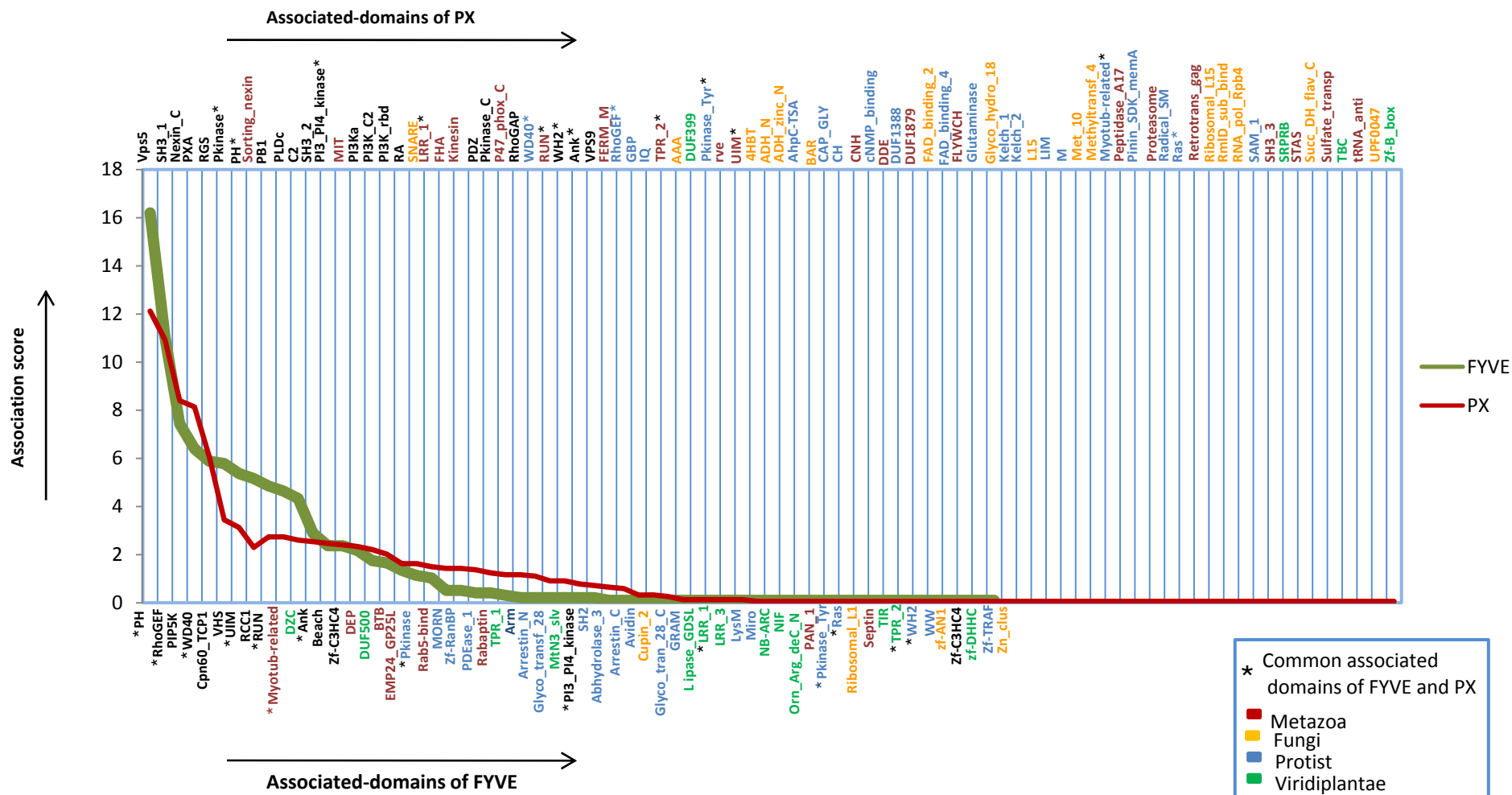

Supplement: Additional file 4 — Association score distribution graph of all FYVE and PX associated domains. The associated domains of FYVE and PX proteins are plotted according to their association score. The upper horizontal axis shows the associated domains of PX and its corresponding graph is drawn in red. The lower horizontal axis is for the associated domains of FYVE and the corresponding graph is drawn in green. Domain names in black font are present in more than one taxonomic group whereas domains that are found only in a particular taxonomic group are coloured according to the colour code given in the figure. [file 1471-2164-11-83-S4.PDF]
